# Evaluation of Awareness of the Need for Orthodontic Treatment Across Different Age Groups in Adolescents and Young Adults

**DOI:** 10.3390/healthcare14203501

**Published:** 2026-10-18

**Authors:** Mevlüde Yüce Polat, Mahmut Tankuş, Başak Akdağ

**Affiliations:** Department of Orthodontics, Faculty of Dentistry, Harran University, 63000 Sanliurfa, Turkey; mahmuttankus@harran.edu.tr (M.T.); basakakdag@harran.edu.tr (B.A.)

**Keywords:** adolescent, awareness, ICON, IOTN, orthodontic treatment need

## Abstract

**Background/Objectives:** This study aimed to determine the awareness levels of individuals presenting to an orthodontic clinic regarding their orthodontic treatment needs and to identify the various factors contributing to the development of this awareness, particularly the developmental period. **Methods:** A total of 160 participants were divided into four equal age groups (*n* = 40 each): early adolescence, middle adolescence, late adolescence, and young adults. Participants’ subjective awareness of orthodontic treatment need was assessed using questionnaire items, the Index of Orthodontic Treatment Need-Aesthetic Component (IOTN-AC), and a Visual Analog Scale (VAS) for self-perceived dental aesthetics, while normative treatment need was evaluated by an expert orthodontist using the IOTN, DAI, and ICON. The primary outcome was aesthetic agreement, classified as underestimation, exact concordance, or overestimation according to the participant-minus-researcher raw IOTN-AC score difference. Sensitivity analyses allowed a ±1-point tolerance and included a pooled analysis of the two youngest age groups. **Results:** Researcher IOTN-AC scores correlated strongly with IOTN-DHC (*ρ* = 0.861; 95% CI 0.804–0.904), DAI (*ρ* = 0.755; 95% CI 0.664–0.827), and ICON (*ρ* = 0.860; 95% CI 0.802–0.903; all Holm-adjusted *p* < 0.001, *n* = 160), confirming construct alignment with normative treatment need. Subjective perception of dental irregularity and skeletal discrepancy increased significantly with age (*p* = 0.005 and *p* < 0.001). In the exploratory pooled model, the odds of underestimation rather than exact concordance were lower at 19–21 years (OR = 0.11; 95% CI 0.04–0.35) and 22–30 years (OR = 0.09; 95% CI 0.03–0.28) than at 12–18 years. The concordant perception group demonstrated higher self-referral and subjective awareness (*p* < 0.05). Nonetheless, these estimates remain susceptible to sparse-data bias. **Conclusions:** Personal awareness of morphological and skeletal discrepancies strengthens with age, with treatment-need underestimation diminishing significantly from age 19 onward in favor of concordant perception. However, conclusions specific to middle adolescence remain uncertain because exact concordance was sparse. While enhancing awareness at younger ages is hypothesized to facilitate timely intervention during active growth periods, longitudinal studies are warranted to evaluate the long-term clinical consequences of early awareness deficits on skeletal growth patterns and subsequent surgical requirements.

## 1. Introduction

Malocclusion, defined as improper relationships of the teeth and/or jaws within themselves or with each other, is the main reason for the need for orthodontic treatment, and orthodontic treatment aims to achieve ideal occlusion [1]. Malocclusions have a prevalence ranging from 39% to 93%, depending on differences in the populations examined [2,3,4]. Not all malocclusions create a treatment need [5]. Therefore, orthodontic treatment need indices have been developed to evaluate the need for orthodontic treatment [6]. Orthodontic treatment need indices aim to classify malocclusions according to treatment necessity [7]. The Index of Orthodontic Treatment Need-Aesthetic Component (IOTN-AC), the IOTN-Dental Health Component (IOTN-DHC), the Dental Aesthetic Index (DAI), and the Index of Complexity, Outcome and Need (ICON) are frequently used when determining treatment need [8,9]. The reliability and validity of these indices were demonstrated when they were introduced [10,11,12].

Individuals who need orthodontic treatment may notice that they have an orthodontic disorder and apply to an orthodontic clinic, or they may be referred by people around them without being aware of their existing malocclusion. Individuals may differ in their ability to recognize and perceive their malocclusions [13,14]. These differences are affected by factors such as aesthetic perception, the degree to which the existing condition is normalized, and psychosocial development [15]. Consequently, heightened awareness of one’s appearance can foster orthodontic treatment awareness and drive subsequent treatment demand [8]. Furthermore, professionally assessed normative thresholds do not always correspond directly with self-perceived orthodontic need, as subjective aesthetic expectations often diverge from researcher-defined parameters [7]. Moreover, the extent to which patient–researcher discordance in treatment need assessment varies across different age groups has not been comprehensively investigated [16]. Individuals’ awareness of their existing malocclusion, the dental-skeletal problem causing malocclusion, and their need for orthodontic treatment is important for seeking orthodontic care and maintaining motivation during treatment [15,16]. Such awareness enables existing orthodontic problems to be treated appropriately and in a timely manner [17]. Therefore, understanding how individuals perceive their orthodontic treatment needs provides valuable clinical context, which has also been discussed in the broader literature as a consideration for healthcare awareness [18,19].

A child’s independent awareness regarding orthodontic treatment is generally considered not to develop until 10–11 years of age [20]. Furthermore, Doğan et al. [8] emphasized that the vast majority of individuals with malocclusion seek orthodontic care primarily driven by aesthetic concerns and self-esteem issues that emerge with the onset of adolescence. In the literature, adolescence is categorized into three distinct stages based on psychosocial development: early adolescence (12–14 years), middle adolescence (15–18 years), and late adolescence (19–21 years) [21]. Although numerous studies have evaluated the awareness of orthodontic treatment need, participants in those investigations have predominantly been evaluated without categorization or grouped without considering developmental criteria [8,9,16,22,23]. To the best of our knowledge, no prior study has investigated how the awareness of orthodontic treatment need is related to or varies across these specific developmental periods. Given this complete absence in the literature, our study aimed to address this fundamental gap by evaluating patients across defined developmental stages rather than treating them as a homogeneous cohort.

The aim of this study was to determine the levels of awareness of orthodontic treatment need among individuals presenting to an orthodontic clinic and the various factors contributing to the development of this awareness, particularly the developmental period. For this purpose, participants were evaluated in four groups based on developmental periods: 12–14 years, 15–18 years, 19–21 years, and 22–30 years. The null hypothesis was that there would be no significant difference in awareness of orthodontic treatment need across developmental periods.

## 2. Materials and Methods

Ethical approval for this study was obtained from the Harran University Non-Interventional Clinical Research Ethics Committee (date: 2 December 2024; decision no.: HRU/25.19.45). A non-probability, sex-stratified quota sampling strategy was employed. Consecutive eligible individuals presenting to the Department of Orthodontics, Faculty of Dentistry, Harran University, for an initial orthodontic examination who met the inclusion criteria and agreed to participate were sequentially enrolled within each of the four age groups until an equal and balanced quota of exactly 20 female and 20 male participants was attained (*n* = 40 per age group; total *N* = 160). This approach was prespecified to control for potential sex-related confounding across age groups; when the target quota for one sex was filled within an age group, further consecutive enrollment of that sex was paused for that stratum, and recruitment proceeded exclusively for the remaining sex until its quota of 20 was fulfilled. The data collection process was completed over nine months (1 January 2025 to 1 October 2025). The inclusion criteria were being literate, being between 12 and 30 years of age, having no systemic disease or previously diagnosed psychological disorder, having no developmental anomaly and/or syndrome, and having no previous orthodontic treatment. Individuals who did not voluntarily agree to participate in the study were not included. Informed voluntary consent forms were obtained from all participants and from the parents of participants under 18 years of age.

Because a child’s own opinion regarding orthodontic treatment does not develop until 10–11 years of age [20], the participants to be included in our study were required to be at least 12 years old. Participants were divided into four groups: 12–14 years (early adolescence), 15–18 years (middle adolescence), 19–21 years (late adolescence), and 22–30 years (young adults). The data collection process continued until an equal number of participants was reached in each group.

**Figure 1:**
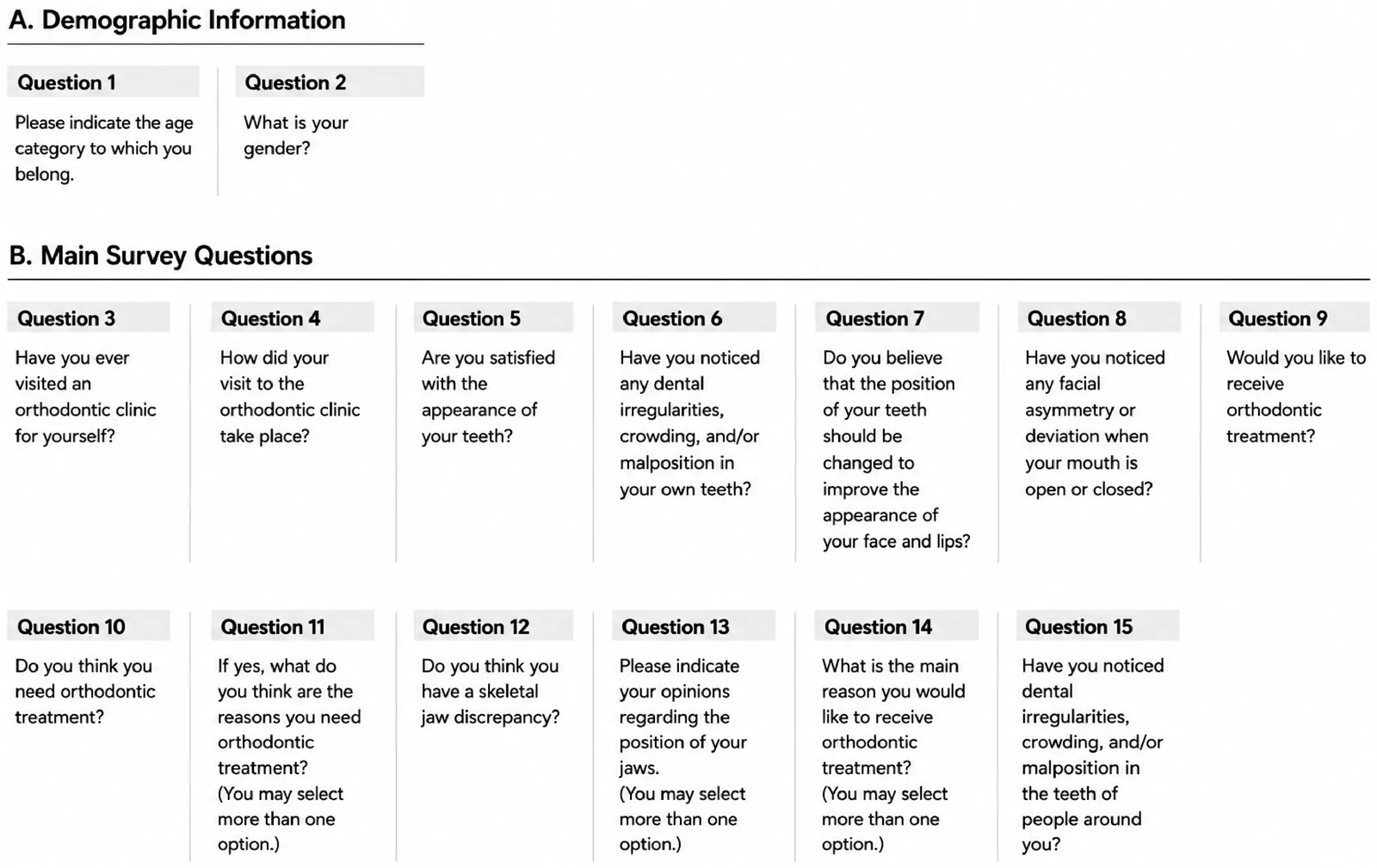
Questions included in the questionnaire form.

In this study, a 15-item questionnaire developed to examine the correlation between developmental periods and awareness of treatment need was used. In addition to the questionnaire, the IOTN-AC and a Visual Analog Scale (VAS) completed by the participants to assess self-perceived dental aesthetics were used. The study also included a participant evaluation form completed independently by the researcher (an expert orthodontist), which incorporated objective orthodontic indices as well as the identical VAS assessed from a professional observer perspective. The content of the questionnaire and participant evaluation form and the methods applied are presented below:

The questionnaire items were adapted from the studies of Doğan et al. [8] and Ertan Erdinç and Erol [9], with face and content validity verified by our research team of orthodontic specialists. Prior to the formal investigation, a preliminary pilot evaluation conducted with 20 participants (excluded from the final sample) confirmed that the questions were clear and easily comprehensible across all age cohorts. Because the questionnaire is structured as a descriptive inventory consisting of discrete categorical variables, conventional internal consistency analyses were not applied. To ensure administration reliability and standardization, all items were administered orally and face-to-face by the same investigator. The questions included in the questionnaire are presented in Figure 1.

To determine subjective orthodontic treatment needs, the IOTN-AC, which enables the subjective evaluation of dental aesthetics, was used [24]. This component, developed by Richmond [25], is based on the rating of 10 intraoral photographs showing the anterior dental region. In this study, the 10 photographs used for the IOTN-AC assessment were presented to the participants simultaneously, and they were asked to select the photograph that most closely resembled their own dental appearance. According to the participant evaluations, the first-grade photograph represents the best dental aesthetics, whereas the tenth-grade photograph represents the worst dental aesthetics [25]. For statistical evaluation of the descriptive treatment-need categories and Cohen’s kappa analysis, and in accordance with established clinical orthodontic thresholds [25,26], the obtained scores were categorized into three standard treatment-need groups: no or slight treatment need (scores 1–4), moderate or borderline treatment need (scores 5–7), and definite or great treatment need (scores 8–10). Scores 1 to 4 were merged into a single category as neither subgroup presents an objective indication for clinically mandated orthodontic intervention. These categories are distinct from the exact raw-score agreement outcome defined below.

VAS is a test method frequently used to evaluate subjective experiences that cannot be measured numerically and consists of a 100 mm horizontal line with verbal descriptors at both ends [17]. Today, it is used to quantitatively assess individuals’ satisfaction with their perception of dental aesthetics. In this study, the 0 mm end of the scale was anchored with the statement “the worst possible dental aesthetics,” and the 100 mm end was anchored with “the most ideal possible dental aesthetics.” Participants were asked to evaluate their dental appearance by placing a vertical mark at the point they considered appropriate. The distance of the marked point from the 0 mm starting point was measured in millimeters and recorded. This evaluation aimed to determine participants’ perceptions of their own dental aesthetics. In addition, the identical 0–100 mm scale was independently marked by the researcher during intraoral clinical examination to provide an expert clinical observer assessment of the participant’s dental aesthetics.

Participants’ perceptions of orthodontic treatment need were evaluated according to their own opinions using the questionnaire items, IOTN-AC, and VAS. The researcher then completed the participant evaluation form by clinically examining participants on the day they presented to the clinic. This form, completed for each participant, aimed to objectively evaluate participants’ need for orthodontic treatment from an expert perspective. This approach was intended to enable an inference regarding participants’ awareness of their need for orthodontic treatment. Accordingly, IOTN, DAI, ICON, and both participant- and researcher-assessed VAS were used in this study to determine participants’ awareness of orthodontic treatment need and to measure their need for orthodontic treatment. In addition, although the identical 0–100 mm scale was independently marked by the researcher during the intraoral clinical examination as an exploratory clinical observer record, this measure was not included in any statistical hypothesis testing or multivariable regression models to prevent analytical redundancy. Normative orthodontic treatment need was established strictly through validated clinical indices. The scales used in addition to the questionnaire completed by the participants and those included in the participant evaluation form completed by the researcher are shown in Table 1.

In addition to the subjective scales (IOTN-AC and VAS) evaluated separately by the participant and the researcher—representing self-perceived aesthetic satisfaction and expert clinical observer assessment, respectively—the purposes and application protocols of the clinical indices used by the researcher to determine normative treatment need are as follows:

The Dental Health Component (DHC), the second component of the IOTN, records the occlusal traits of malocclusion [24]. In this component, the primary objective is to define the existing malocclusion based on the most prominent and worst occlusal feature of the individual being assessed [24]. Evaluations were conducted hierarchically according to the standardized criteria of Richmond et al. [26], assessing criteria such as missing teeth, overjet, reverse overjet, crossbite, contact point displacement, open bite, and deep overbite. Although Grade 1 in the original classification represents no treatment need (minor irregularities with contact point displacement <1 mm), it was not retained as an active category because Grade 1 was not identified among the 160 participants. Participants were categorized into four distinct treatment need groups as follows:

Low (Grade 2): Increased overjet 3.5–6 mm with competent lips, reverse overjet 0.1–1 mm, contact point displacement 1–2 mm, open bite 1–2 mm, deep overbite ≥3.5 mm without gingival contact, or discrepancy in occlusal relationship up to half a unit.

Borderline (Grade 3): Increased overjet 3.5–6 mm with lip incompetence, reverse overjet 1–3.5 mm, crossbite with 1–2 mm discrepancy, contact point displacement 2–4 mm, open bite 2–4 mm, or non-traumatic deep overbite.

High (Grade 4): Less extensive hypodontia, increased overjet 6–9 mm, reverse overjet >3.5 mm, crossbite with >2 mm discrepancy, contact point displacement >4 mm, open bite >4 mm, traumatic deep overbite, partially erupted/tipped teeth, or supernumerary teeth. Very high (Grade 5): Extensive hypodontia requiring pre-restorative orthodontics, increased overjet >9 mm, reverse overjet >3 mm with masticatory and speech difficulties, cleft lip/palate or other craniofacial anomalies, submerged deciduous teeth, or impacted permanent teeth due to pathological reasons (excluding third molars) [26].

ICON is a five-component index that determines treatment complexity, outcome, and need [12,26,27,28,29]. The index was applied through clinical examination of each participant along with the evaluation of the Aesthetic Component (AC) of the IOTN. The five components were assessed according to their scoring protocols and validated weights as follows [12]:

Aesthetic Component (weight 7): Scored from 1 to 10 using the IOTN-AC photographic scale.

Upper arch crowding/spacing (weight 5): Crowding was scored as 0 (<2 mm), 1 (2.1–5 mm), 2 (5.1–9 mm), 3 (9.1–13 mm), 4 (13.1–17 mm), and 5 (>17 mm or impacted tooth); spacing was scored as 0 (<2 mm), 1 (2.1–5 mm), 2 (5.1–9 mm), and 3 (>9 mm).

Crossbite (weight 5): Scored as 0 (absent) or 1 (present).

Anterior open bite/deep overbite (weight 4): Scored as 0 (complete overlap), 1 (<1 mm), 2 (1.1–2 mm), 3 (2.1–4 mm), and 4 (>4 mm).

Buccal segment anteroposterior relationship (weight 3): Scored as 0 (cusp-to-fossa relationship; Class I, II, or III), 1 (intermediate relationship between cusp-to-fossa and cusp-to-cusp), and 2 (cusp-to-cusp relationship).

The score obtained for each component was multiplied by its predetermined weight, and the products were summed to calculate the total ICON score. Based on participant data, if the total score obtained was equal to or greater than 43 (≥43), the individual was considered to need orthodontic treatment according to the index [12].

DAI, an index with 10 occlusal characteristics, has been shown in previous studies to have an important relationship with the perception of treatment need [11,30]. In this study, the Turkish version of the index tested by Karaağaç and Küçükeşmen [31] was used. The 10 occlusal traits and their regression weights were clinically evaluated: missing incisors, canines, and premolars in the dental arch (weight 6); crowding in the incisal segment (0 = no crowding, 1 = one segment, 2 = two segments; weight 1); spacing in the incisal segment (0 = no spacing, 1 = one segment, 2 = two segments; weight 1); midline diastema in millimeters (weight 3); largest anterior maxillary irregularity in millimeters (weight 1); largest anterior mandibular irregularity in millimeters (weight 1); anterior maxillary overjet in millimeters (weight 2); anterior mandibular overjet in millimeters (weight 4); vertical anterior open bite in millimeters (weight 4); and anteroposterior molar relationship (0 = normal, 1 = 1/2 cusp mesial/distal, 2 = one full cusp or more mesial/distal; weight 3) [30]. The score obtained for each component was multiplied by its regression weight, the products were summed, and a constant value of 13 was added to calculate the final DAI score [30]:

Participants were categorized into four treatment need groups according to the total score obtained [30]:

No or slight treatment need (Score ≤ 25);

Elective treatment need (Score 26–30);

Severe treatment need (Score 31–35);

Very severe treatment need (Score ≥ 36).

### Participant–Researcher Agreement Assessment

To evaluate the agreement between participant self-perception and researcher assessment, the IOTN-AC raw-score difference was calculated as:

ΔAC = participant IOTN-AC raw score − researcher IOTN-AC raw score

Based on this score difference, agreement was operationalized into three distinct categories: underestimation (ΔAC < 0), exact concordance (ΔAC = 0), and overestimation (ΔAC > 0). Concordance required identical raw scores rather than merely falling into the same broad treatment-need tier, and no tolerance interval was applied. Both participant and researcher raw scores were complete and within the allowable 1–10 range for all 160 participants, with no missing, invalid, or unclassifiable data. To evaluate whether the researcher’s aesthetic perception reliably tracked clinical severity, researcher raw IOTN-AC scores were benchmarked against established objective orthodontic indices (IOTN-DHC, DAI, and ICON). However, because exact matching represents a stringent operational criterion and a minor one-point deviation on a 10-point visual scale does not necessarily indicate a clinically meaningful perceptual discrepancy, an exploratory threshold sensitivity analysis was also conducted to evaluate the robustness of categorical agreement. Under this secondary definition, concordance was operationalized by allowing a ±1-point tolerance margin (−1 ≤ ΔAC ≤ 1), whereas underestimation was defined as ΔAC < −1 and overestimation as ΔAC > 1.

## 3. Statistical Analysis

Sample size was estimated a priori using G*Power 3.1 for the primary analysis comparing treatment-need awareness agreement across four age groups and three awareness categories. Based on previous IOTN/AC-IOTN-based orthodontic awareness studies [7,15], a conservative medium effect size was assumed for the χ^2^ test of independence (Cohen’s w = 0.30; df = 6), with α = 0.05 and 80% power, yielding a minimum required sample size of 152 participants. After allowing for approximately 5% incomplete or unusable data, the target sample size was increased to 160, which was achieved in the final analytical cohort (comprising exactly 40 participants per age cohort, balanced equally with 20 females and 20 males through stratified quota recruitment).

The original analyses were reported using IBM SPSS Statistics for Windows, version 27.0 (IBM Corp., Armonk, NY, USA). The revised analyses were performed in Python 3.12.14 using SciPy 1.17.0 and statsmodels 0.15.0. All tests were two-tailed, and statistical significance was set at *p* < 0.05. The main comparison variable of the study was age group based on developmental period, and participants were analyzed in four groups: 12–14 years, 15–18 years, 19–21 years, and 22–30 years. In addition, the primary outcome of the study was treatment-need awareness agreement, defined according to the difference between the participant and researcher IOTN-AC assessments, and this variable was analyzed in three categories: underestimation of treatment need, concordant perception, and overestimation of treatment need. Categorical variables were presented as numbers and percentages [*n* (%)] and as *n*/N (%) when denominators varied. The distributions of continuous variables were evaluated using graphical methods and assessment of normality; normally distributed variables were reported as mean ± standard deviation (SD), whereas non-normally distributed or ordinal variables were reported as median [interquartile range, IQR].

The Fisher–Freeman–Halton test was used whenever any expected count was below 5; Pearson’s chi-square test was used otherwise. The categorical comparisons were verified using the same fixed-margin framework. Fixed-margin probabilities were enumerated for analyses involving two categories. For analyses with more than two categories in both dimensions, two-sided Fisher–Freeman–Halton *p* values were estimated by Monte Carlo sampling of 1,000,000 fixed-margin tables (seed 20261001), using probability ordering; these are explicitly labeled as Monte Carlo estimates. Cramer’s *V* was calculated from the Pearson chi-square statistic as a descriptive effect size, regardless of the method used to obtain the *p* value.

The participant VAS score was summarized as mean ± SD across age groups and compared using one-way ANOVA. The IOTN-AC raw-score difference and VAS comparisons across agreement groups were summarized as median [Q1, Q3] and analyzed with the Kruskal–Wallis test. Dunn pairwise comparisons were adjusted using the Bonferroni method across all pairs within each outcome. The ANOVA effect size was eta-squared (η^2^ = between-group sum of squares/total sum of squares), whereas the Kruskal–Wallis effect size was rank epsilon-squared (ε^2^ = H/[*N* − 1]). These are different measures and are labeled accordingly. Unweighted Cohen’s kappa summarized agreement between the three treatment-need categories and did not define the primary raw-score agreement groups. Researcher-assessed VAS was excluded from statistical hypothesis testing and multivariable modeling to prevent analytical redundancy.

Benjamini–Hochberg false discovery rate adjustment was applied separately across distinct comparison families for the subgroup analyses, using a false discovery rate *q* value below five percent for interpretation within each family. The single primary age-group comparison of raw-score agreement was not multiplicity-adjusted, and the overall omnibus comparison was evaluated as a single test. The remaining secondary comparisons across categories and the regression analyses were exploratory; their unadjusted *p* values are reported and should not be interpreted as confirmatory evidence.

To limit model complexity given the sparse outcome categories, the multinomial model included age group and sex, with concordant perception as the reference outcome, 12–14 years as the reference age group, and female sex as the reference. The model estimated four non-intercept coefficients per outcome contrast. Participant VAS was excluded because it measures a related subjective perception; researcher IOTN-AC category was excluded because the researcher score contributes directly to the outcome definition. Previous attendance was omitted to reduce model complexity and because its relationship with age and agreement could not be resolved temporally in this cross-sectional study. An age-group-only model was fitted as a sensitivity analysis. No stepwise or significance-based variable selection was used. Odds ratios (ORs), Wald 95% confidence intervals (CIs), and two-sided *p* values are reported. Both models were exploratory.

In the threshold sensitivity analysis (±1-point tolerance), the age-group distribution was compared using a probability-ordered Fisher–Freeman–Halton Monte Carlo test with 1,000,000 fixed-margin samples (seed: 20261009). A plus-one correction was applied to the simulated *p* value, and its Monte Carlo uncertainty was summarized using a binomial 95% CI. Cramer’s *V* was derived from Pearson’s chi-square statistic. No multinomial model was fitted under this definition because the overestimation category contained only five participants and included a zero age-group cell. To address sparse exact concordance in the 15–18-year group, the 12–14- and 15–18-year groups were combined for a separate sensitivity analysis, retaining the exact raw-score outcome. The pooled 12–18-year group (*n* = 80) was compared with the 19–21- and 22–30-year groups (*n* = 40 each). The categorical comparison used 1,000,000 fixed-margin Monte Carlo samples (seed 20261010), with the same probability ordering and uncertainty calculation. The age-group-and-sex multinomial model was refitted with 12–18 years as the age reference, female sex as the sex reference, and exact concordance as the outcome reference. This model estimated three slope coefficients per contrast (eight parameters including two intercepts).

To benchmark researcher IOTN-AC ratings against objective clinical indices, Spearman rank correlations were calculated between the researcher raw IOTN-AC scores and IOTN-DHC grade, DAI total score, and ICON total score (*n* = 160). Percentile 95% confidence intervals were derived using 20,000 paired bootstrap resamples for each comparison (seeds 20261018–20261020, respectively). Two-sided asymptotic *p* values were Holm-adjusted across these three correlations. To prevent artificial dichotomization artifacts and classification discrepancies, the relationship between the researcher’s scores and the objective indices was directly benchmarked using continuous scores and recorded grades via Spearman rank correlation. Rather than assuming absolute diagnostic interchangeability, these analyses evaluate the ranking consistency and the strength of construct alignment between the researcher’s aesthetic grading and established normative treatment-need criteria.

## 4. Results

The sample consisted of 160 volunteer participants who presented to the Harran University Orthodontics Clinic. Of the participants, 50% were female (*n* = 80) and 50% were male (*n* = 80). The distribution of participants according to age groups was fully balanced, with 20 female and 20 male participants in each group; this balance resulted from the prespecified quota sampling strategy, and the sex ratio was therefore identical across cohorts (*p* = 1.000).

**Figure 2:**
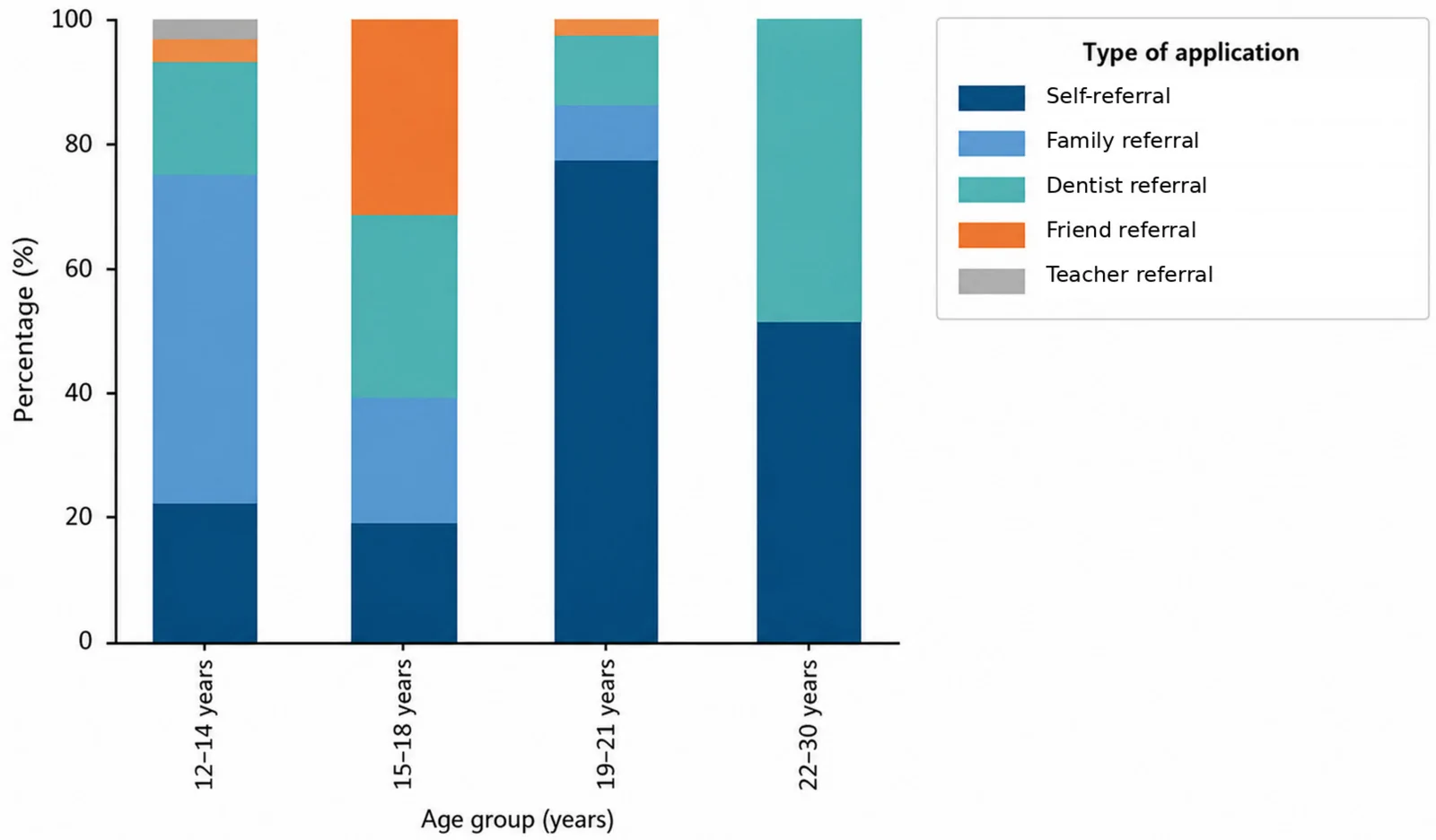
Distribution of the mode of application to the orthodontic clinic according to age groups.

Previous orthodontic clinic application history differed significantly across age groups; this rate was 12.5% in the 12–14 and 15–18 age groups, increased to 40.0% in the 19–21 age group, and reached 57.5% in the 22–30 age group (*p* < 0.001; Cramer’s *V* = 0.416). The mode of application to the clinic also changed markedly with age (Fisher–Freeman–Halton Monte Carlo *p* < 0.001; Cramer’s *V* = 0.446). In the early age group, family referral was predominant (55.0% for the 12–14 age group), whereas self-referral was prominent in the 19–21 age group (77.5%); in the 22–30 age group, self-referral (52.5%) and dentist referral (47.5%) were the main modes of application (Table 2 and Figure 2).

The proportion of participants noticing irregularity in their own teeth increased significantly with age; this rate was 72.5% in the 12–14 age group and 77.5% in the 15–18 age group, whereas it was 95.0% in the 19–21 and 22–30 age groups (*p* = 0.005; Cramer’s *V* = 0.284). The proportion of participants who perceived having a skeletal jaw discrepancy varied significantly across age groups; this rate was 12.5% in the 12–14-year group and 20.0% in the 15–18-year group, compared with 47.5% in the 19–21-year group and 43.6% in the 22–30-year group (*p* < 0.001; Cramer’s *V* = 0.324). The rate at which others noticed participants’ dental irregularity increased markedly with age. This rate was 25.0% in the 12–14 age group, whereas it was 87.5% in the 19–21 age group and 92.5% in the 22–30 age group (*p* < 0.001; Cramer’s *V* = 0.588) (Supplementary Table S1). In contrast, no statistically significant differences were found across age groups for the other subjective evaluations shown in Supplementary Table S1 (*p* > 0.05).

Among participants who stated “I think orthodontic treatment is necessary” (*n* = 151), raw comparisons of reasons suggesting the necessity of orthodontic treatment showed differences between age groups for crowding (*p* = 0.024), crooked teeth (*p* = 0.002), some teeth being positioned higher (*p* = 0.048), upper anterior teeth positioned forward (*p* = 0.024), presence of supernumerary teeth (*p* = 0.016), and dissatisfaction with facial appearance (*p* = 0.046). After Benjamini–Hochberg correction, the strongest variable that maintained statistical robustness was crooked teeth (*q* = 0.018). An increasing trend with age was notable for crowding; the rate increased from 43.2% in the 12–14 age group to 76.3% in the 22–30 age group. Dissatisfaction with facial appearance was particularly prominent in the 19–21 age group, reaching 50.0% (Supplementary Table S2).

Participants’ perceptions of jaw position differed significantly across age groups (Fisher–Freeman–Halton test, Monte Carlo *p* < 0.001; Cramer’s *V* = 0.301). The response “I have no idea” was highest in the 12–14 age group at 60.0%, whereas it decreased to 10.0% in the 19–21 age group. In contrast, the “normal” assessment increased with age and reached 45.0% in the 19–21 and 22–30 age groups. More specific skeletal evaluations varied across cohorts; the perception that “my upper jaw is forward” was most frequently reported in the 15–18 age group (22.5%), whereas reports of “my lower jaw is backward” were most prominent in the 22–30 age group (17.5%). Because more than one response was permitted, the observed combination “upper jaw forward and lower jaw backward” was retained as a separate category in the omnibus comparison (Supplementary Table S3).

Overall, 79.4% of participants (*n* = 127/160) expressed a desire to receive orthodontic treatment (answered “yes” to the question “Would you like to receive orthodontic treatment?”), with cohort-specific rates of 80.0% (32/40), 85.0% (34/40), 85.0% (34/40), and 67.5% (27/40) across the 12–14, 15–18, 19–21, and 22–30 age groups, respectively. Among participants who wanted orthodontic treatment (*n* = 127), dissatisfaction with dental appearance was the most frequent source of motivation in all age groups, ranging from 78.1% to 94.1%. The reason “Desire to breathe comfortably” differed significantly across age groups (*p* = 0.002; *q* = 0.008; Cramer’s *V* = 0.336); this reason was particularly prominent in the 15–18-year (35.3%) and 22–30-year (25.9%) groups. Environmental referral was 37.5% in the 12–14 age group and 50.0% in the 15–18 age group, whereas it decreased below 10% in the 19–21 and 22–30 age groups (Supplementary Table S4). In contrast, no statistically significant difference was found between age groups in terms of the other reasons for wanting orthodontic treatment in Supplementary Table S4 (*p* > 0.05).

Table 3 demonstrates the main methodological distinction of the study. The participant IOTN-AC category differed significantly between age groups (*p* < 0.001; Cramer’s *V* = 0.357). In participant assessments, the ‘no or slight need (1–4)’ category was observed at high rates of 62.5% in the 12–14 age group and 82.5% in the 15–18 age group, whereas this rate decreased to 25.0% in the 19–21 and 22–30 age groups. In contrast, perceptions of ‘moderate/borderline need (5–7)’ and ‘definite/great need (8–10)’ were observed more frequently in older age groups. No significant differences were detected in orthodontic treatment need indicators evaluated by the researcher (*p* > 0.05) (Table 3).

**Table 3:** Comparison of objective and subjective orthodontic treatment need indicators according to age groups.

Variable	12–14 Years	15–18 Years	19–21 Years	22–30 Years	Total	*p*	Effect Size
Participant Index of Orthodontic Treatment Need-Aesthetic Component (IOTN-AC) category						<0.001	Cramer’s *V* = 0.357
└ No treatment need (1–4)	25 (62.5)	33 (82.5)	10 (25.0)	10 (25.0)	78 (48.8)		
└ Borderline need (5–7)	9 (22.5)	6 (15.0)	22 (55.0)	23 (57.5)	60 (37.5)		
└ High need (8–10)	6 (15.0)	1 (2.5)	8 (20.0)	7 (17.5)	22 (13.8)		
Researcher IOTN-AC category						0.972	Cramer’s *V* = 0.065
└ No treatment need (1–4)	4 (10.0)	5 (12.5)	4 (10.0)	6 (15.0)	19 (11.9)		
└ Borderline need (5–7)	19 (47.5)	19 (47.5)	21 (52.5)	21 (52.5)	80 (50.0)		
└ High need (8–10)	17 (42.5)	16 (40.0)	15 (37.5)	13 (32.5)	61 (38.1)		
Index of Orthodontic Treatment Need-Dental Health Component (IOTN-DHC) grade						0.976	Cramer’s *V* = 0.076
└ Low	4 (10.0)	6 (15.0)	7 (17.5)	6 (15.0)	23 (14.4)		
└ Borderline	17 (42.5)	16 (40.0)	18 (45.0)	15 (37.5)	66 (41.2)		
└ High	14 (35.0)	12 (30.0)	12 (30.0)	15 (37.5)	53 (33.1)		
└ Very high	5 (12.5)	6 (15.0)	3 (7.5)	4 (10.0)	18 (11.2)		
Treatment need according to Index of Complexity, Outcome and Need (ICON)						0.822	Cramer’s *V* = 0.076
└ No (<43)	6 (15.0)	4 (10.0)	4 (10.0)	6 (15.0)	20 (12.5)		
└ Yes (≥43)	34 (85.0)	36 (90.0)	36 (90.0)	34 (85.0)	140 (87.5)		
Dental Aesthetic Index (DAI) category						0.506	Cramer’s *V* = 0.136
└ No or slight treatment need (Score ≤ 25)	7 (17.5)	4 (10.0)	2 (5.0)	2 (5.0)	15 (9.4)		
└ Elective treatment need (Score 26–30)	12 (30.0)	15 (37.5)	19 (47.5)	18 (45.0)	64 (40.0)		
└ Severe treatment need (Score 31–35)	9 (22.5)	8 (20.0)	10 (25.0)	12 (30.0)	39 (24.4)		
└ Very severe treatment need (Score ≥ 36)	12 (30.0)	13 (32.5)	9 (22.5)	8 (20.0)	42 (26.2)		

Data are *n* (%). Pearson’s chi-square test was used for participant IOTN-AC category and ICON. Researcher IOTN-AC category, IOTN-DHC grade, and DAI category were compared using Fisher–Freeman–Halton tests with Monte Carlo estimation (1,000,000 samples; seed 20261001). Cramer’s *V* is based on Pearson’s chi-square statistic. Treatment-need categories are reported as recorded. These secondary *p* values are unadjusted. This table presents objective treatment need indices and subjective treatment need indicators within the same framework; the main agreement/disagreement analysis is given separately in Table 4.

To benchmark the researcher’s aesthetic perception against normative treatment need, researcher raw IOTN-AC scores were evaluated against objective clinical indices. Researcher raw IOTN-AC scores demonstrated strong and statistically significant positive rank correlations with IOTN-DHC grade (*ρ* = 0.861; 95% CI 0.804–0.904), DAI total (*ρ* = 0.755; 95% CI 0.664–0.827), and ICON total (*ρ* = 0.860; 95% CI 0.802–0.903; all Holm-adjusted *p* < 0.001; Supplementary Table S5). All comparisons included 160 participants.

**Figure 3:**
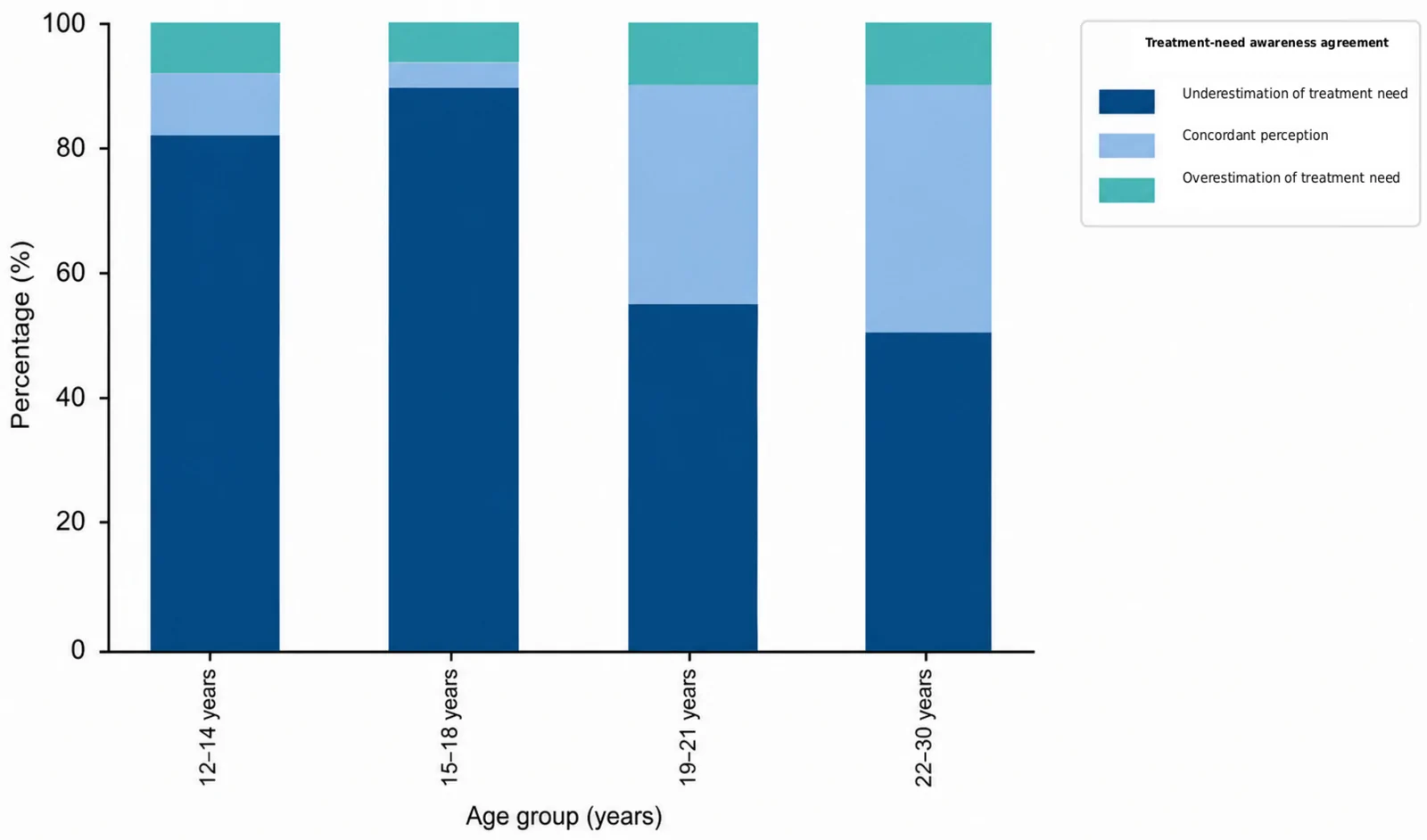
Raw-score agreement between participant and researcher IOTN-AC ratings across age groups (*n* = 40 per group). Underestimation, exact concordance, and overestimation denote ΔAC < 0, ΔAC = 0, and ΔAC > 0, respectively, where ΔAC is participant minus researcher raw IOTN-AC score.

**Figure 4:**
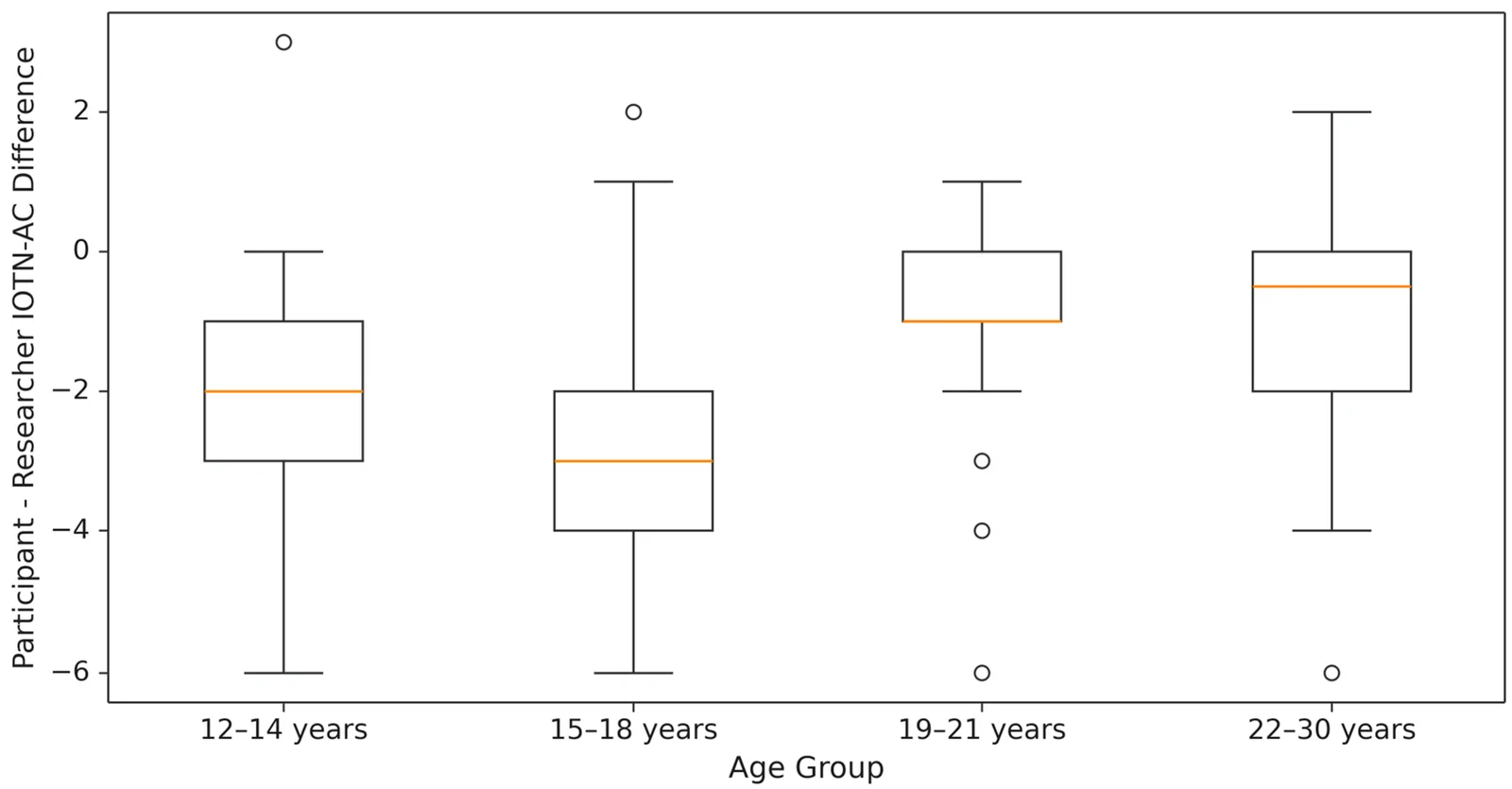
Distribution of the participant–researcher raw IOTN-AC difference according to age groups.

Table 4 is the primary analysis table of the study. Treatment-need awareness agreement differed significantly between age groups (Fisher–Freeman–Halton Monte Carlo *p* < 0.001; Cramer’s *V* = 0.283). Underestimation of treatment need, defined as the participant assessing treatment need as milder than the researcher, was the dominant pattern in the 12–14 age group (82.5%) and 15–18 age group (90.0%), whereas this rate decreased to 55.0% in the 19–21 age group and 50.0% in the 22–30 age group (Table 4). This distribution is shown in Figure 3. The participant–researcher raw IOTN-AC difference also supported this pattern; the median difference was −2.0 [−3.0 to −1.0] in the 12–14 age group, −3.0 [−4.0 to −2.0] in the 15–18 age group, −1.0 [−1.0 to 0.0] in the 19–21 age group, and −0.5 [−2.0 to 0.0] in the 22–30 age group (H = 40.023; *p* < 0.001; ε^2^ = 0.252). The box plot in Figure 4 shows that younger age groups exhibited a more negative distribution of differences. In the threshold sensitivity analysis allowing a ±1-point tolerance in IOTN-AC raw scores (−1 ≤ ΔAC ≤ 1), overall concordance increased from 35/160 (21.9%) to 77/160 (48.1%); 78 participants (48.8%) remained below the researcher’s score by more than one point, and five (3.1%) were above it by more than one point. Concordance under this definition was 10/40 (25.0%), 7/40 (17.5%), 32/40 (80.0%), and 28/40 (70.0%) across the four age cohorts, respectively. The statistically significant pattern across age groups observed without a tolerance interval also persisted under a ±1-point tolerance margin (Fisher–Freeman–Halton Monte Carlo *p* < 0.001; Cramer’s *V* = 0.395; Supplementary Table S6).

**Table 4:** Analysis of awareness agreement between participant and researcher IOTN-AC assessments according to age groups.

Variable	12–14 Years	15–18 Years	19–21 Years	22–30 Years	Total	*p*	Effect Size	Post Hoc
Treatment-need awareness agreement						<0.001	Cramer’s *V* = 0.283	
└ Underestimation of treatment need	33 (82.5)	36 (90.0)	22 (55.0)	20 (50.0)	111 (69.4)			
└ Concordant perception	4 (10.0)	1 (2.5)	14 (35.0)	16 (40.0)	35 (21.9)			
└ Overestimation of treatment need	3 (7.5)	3 (7.5)	4 (10.0)	4 (10.0)	14 (8.8)			
Participant–researcher raw IOTN-AC difference, median [IQR]	−2.0 [−3.0–1.0]	−3.0 [−4.0–2.0]	−1.0 [−1.0–0.0]	−0.5 [−2.0–0.0]	−1.0 [−3.0–0.0]	<0.001	ε^2^ = 0.252	12–14 vs. 19–21: *p* = 0.002; 12–14 vs. 22–30: *p* = 0.003; 15–18 vs. 19–21: *p* < 0.001; 15–18 vs. 22–30: *p* < 0.001
Cohen kappa	0.055	−0.011	0.468	0.627	0.252	—	—	

Data are *n* (%) or median [Q1, Q3]. ΔAC = participant minus researcher raw IOTN-AC score; underestimation: ΔAC < 0; concordance: ΔAC = 0; overestimation: ΔAC > 0. The categorical omnibus *p* value is a Fisher–Freeman–Halton Monte Carlo estimate. Raw-score differences were compared using Kruskal–Wallis and Dunn–Bonferroni tests. Rank ε^2^ = H/(*N* − 1). Kappa is unweighted agreement between the three recorded treatment-need categories, not exact raw-score concordance. All analyses include 160 participants. IQR, interquartile range; ε^2^, epsilon-squared.

**Figure 5:**
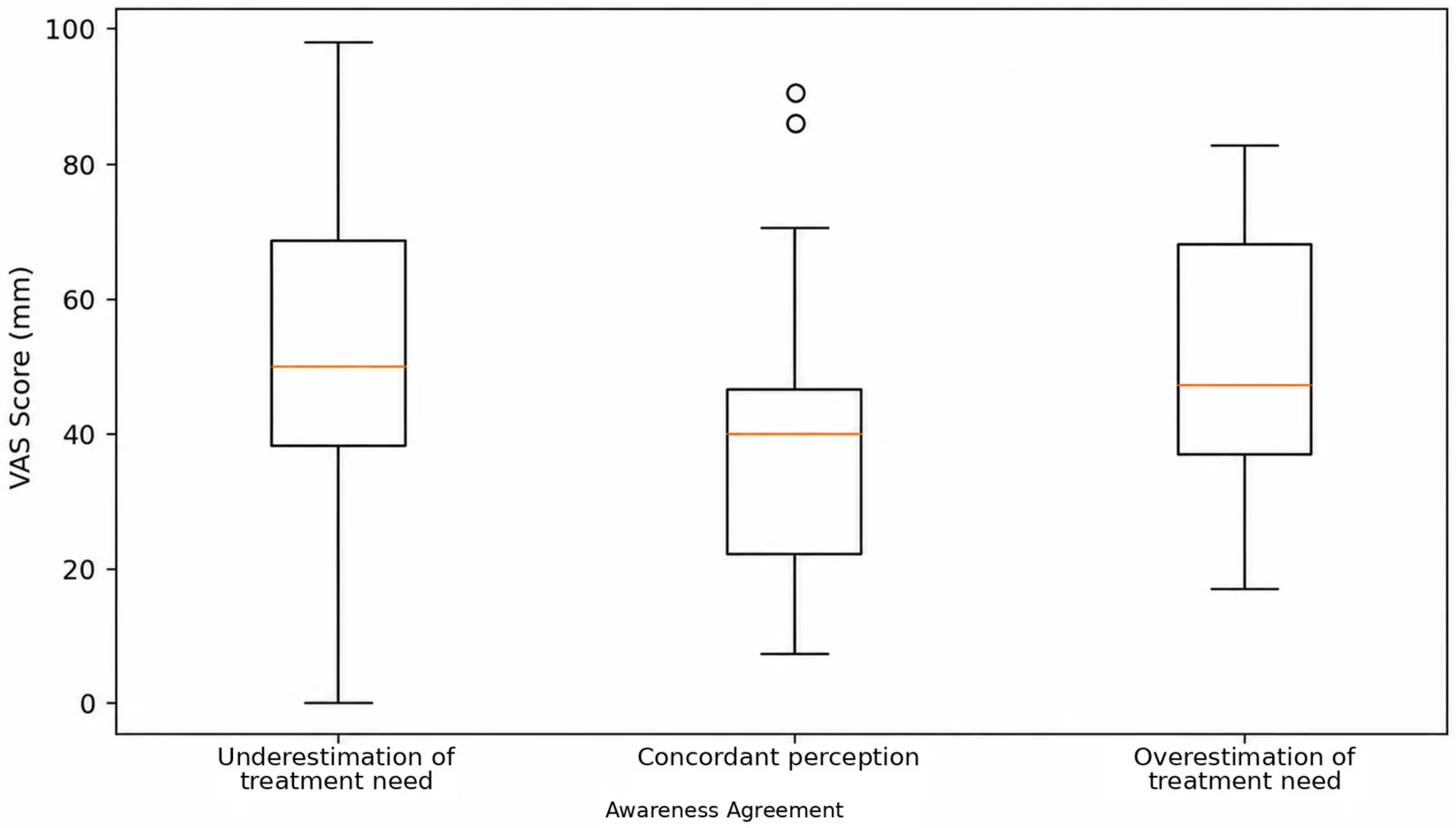
Distribution of participant-assessed VAS score according to treatment-need awareness agreement. Underestimation, exact concordance, and overestimation denote ΔAC < 0, ΔAC = 0, and ΔAC > 0, respectively, where ΔAC is participant minus researcher raw IOTN-AC score.

Clinical application patterns according to treatment-need awareness agreement categories are shown in Supplementary Table S7. When these patterns were examined, previous orthodontic clinic application history was higher in the concordant perception group (48.6%; *p* = 0.038). Self-referral was also highest in the concordant perception group, at 68.6% (Monte Carlo *p* < 0.001). In contrast, family referral was more prominent in the underestimation group (27.9%; *p* < 0.001). Noticing irregularity in one’s own teeth reached 97.1% in the concordant perception group (*p* = 0.026). Thinking that one had a skeletal jaw discrepancy was also significantly higher in the concordant perception group (60.0%; *p* < 0.001). The rate of others noticing dental irregularity reached the highest level in the concordant perception group, at 91.4% (*p* < 0.001; Cramer’s *V* = 0.335) (Supplementary Table S7). The participant-assessed VAS score also differed significantly across awareness groups; the median VAS was 50.0 [38.5–69.0] in the underestimation group, 40.0 [22.0–46.5] in the concordant perception group, and 47.0 [37.2–68.2] in the overestimation group (*p* = 0.007; ε^2^ = 0.062). Figure 5 shows that the participant-assessed VAS score tended to be lower, particularly in the concordant perception group, and shifted toward higher values in the underestimation group. Therefore, awareness agreement may be associated not only with age, but potentially also with subjective aesthetic assessment and clinic-attendance behaviors.

In the reduced multinomial model adjusted for sex, the odds of underestimation rather than concordant perception were lower in the 19–21-year group (OR = 0.19; 95% CI 0.06–0.65; *p* = 0.008) and the 22–30-year group (OR = 0.15; 95% CI 0.04–0.52; *p* = 0.003) than in the 12–14-year group (Table 5). No clear age-group associations were demonstrated for overestimation, and its confidence intervals were wide. The age-group-only sensitivity model produced similar underestimation estimates (OR = 0.19 and 0.15, respectively; Supplementary Table S8). Under the ±1-point threshold sensitivity definition, multinomial logistic regression was not fitted because the overestimation category comprised only five participants and included a zero age-group cell, precluding model estimation.

After combining the two youngest groups, exact concordance was observed in 5/80 participants aged 12–18 years (6.3%), compared with 14/40 aged 19–21 years (35.0%) and 16/40 aged 22–30 years (40.0%). The categorical difference persisted (Monte Carlo *p* < 0.001; Cramer’s *V* = 0.280). In the exploratory sex-adjusted pooled model, the odds of underestimation rather than exact concordance were lower at 19–21 years (OR = 0.11; 95% CI 0.04–0.35; *p* < 0.001) and 22–30 years (OR = 0.09; 95% CI 0.03–0.28; *p* < 0.001) than at 12–18 years (Supplementary Table S9). All model estimates remain susceptible to sparse-data bias, and overestimation estimates were imprecise. The categorical comparison describes an age-group difference but does not establish a distinct effect in the 15–18-year group.

## 5. Discussion

Orthodontic treatment need indices are measurement tools used to systematically determine the extent to which malocclusion deviates from ideal occlusion from subjective and objective perspectives [6]. A review of the literature shows that studies have objectively evaluated orthodontic treatment need in different populations [22,32,33,34]. However, individuals’ awareness of their need for this treatment is as clinically important as the need for treatment itself. In this context, enhancing informational guidance for individuals regarding malocclusion is hypothesized to support timely consultation and appropriate access to care.

In this study, orthodontic treatment need indices were used to evaluate the awareness of individuals presenting to an orthodontic clinic regarding their orthodontic treatment needs, together with the aesthetic, morphological, functional, and social factors contributing to the development of this awareness, with age group as the primary factor of interest.

Jasim et al. [7] examined awareness of orthodontic treatment need in a manner similar to our study. However, in that study, participants were evaluated as a single group aged 11–14 years without considering developmental periods. In their cohort, Jasim et al. [7] demonstrated that adolescents frequently underestimate their treatment needs compared with expert evaluations; using the IOTN-AC, adolescents reported definite treatment need at nearly half the rate diagnosed by orthodontists (4.8% vs. 8.5%, respectively). Consistent with those findings, participants in our 12–14 age group similarly exhibited a marked tendency to underestimate their treatment need, indicating consistency between those studies and our youngest cohort. Furthermore, because age grouping in our study was designed around distinct developmental periods, we demonstrated that exact participant–researcher concordance was more frequent in the two groups aged 19 years and older. Although exact concordance was observed more frequently from age 19 onward, these observations remain constrained by subgroup sample sizes and sparse-data limitations, and this between-group cross-sectional comparison may not necessarily demonstrate a longitudinal increase within individuals over time.

Some studies have shown that dental aesthetic perception increases with age and that awareness differs significantly across age groups [15,22,35,36,37]. Bos et al. [36] reported that female patients aged 13–16 years reported more complaints about their malocclusions than patients of other ages. In a study by Aikins et al. [35], awareness of orthodontic treatment need was evaluated among individuals aged 12–18 years, and awareness of treatment need was found to be higher in older children (16–18 years). In a study by Zhao et al. [15], a relationship was established between individuals’ awareness of orthodontic treatment need and desire to receive orthodontic treatment. However, that study examined two groups, children and adults, and did not examine orthodontic treatment need awareness across developmental periods. Similarly, in a recent study, participants were divided into only two groups—children and adults—and a statistically significant discordance between patient and researcher assessments was reported in both cohorts [16]. Notably, that study indicated that this perceptual gap did not diminish or improve with age, but rather persisted across both groups. Similarly, Sultana and Hossain [22] evaluated a single group aged 11–15 years without considering developmental periods and did not report a significant finding regarding perceived orthodontic treatment need. In contrast, by evaluating four distinct developmental stages, our study demonstrated that this discrepancy decreases markedly from late adolescence onward, as participant self-assessments align with researcher ratings—which were also found to be consistent with objective treatment need indices—resulting in a significant increase in participant–researcher perceptual agreement.

Taibah and Al-Hummayani [23], in a study with two age groups, 12–15 years and 16–19 years, reported a statistically significant but weak relationship between subjective and normative needs in both age groups and concluded that there was a lack of awareness of orthodontic treatment need. This finding is partially inconsistent with the findings of our study. Our study supports the findings of Taibah and Al-Hummayani [23] for younger age groups but shows the opposite pattern in older age groups. This discrepancy may be because only the IOTN-AC scale was used in the study by Taibah and Al-Hummayani [23] to evaluate participants’ awareness of orthodontic treatment need.

In a study conducted by Erdinç and Erol [9] among participants aged 13–20 years, the authors reported that most individuals who self-referred to an orthodontic clinic were high school students. This is partially inconsistent with the findings of our study. Our findings are consistent with those of Erdinç and Erol [9] for younger age groups but show the opposite pattern in older age groups. This discrepancy may be due to the imbalance in the distribution of sample groups in that study. In our study, this limitation was addressed by maintaining equal sample sizes across age groups, thereby increasing the statistical power of between-group comparisons.

In a study by Doğan et al. [8], awareness of irregularity in one’s own teeth was examined among patients aged 15–33 years who presented to an orthodontic clinic. However, no scale was used to objectively test awareness. Therefore, participants’ responses allowed only a subjective interpretation. In addition, the study found that patients presented to the orthodontic clinic following dentist referral rather than as a result of their own awareness. This finding contrasts with the results of our study. This may be due to the small sample size of that study and the uneven age distribution, because 88.1% of the sample in that study was in the 15–19 age range. In our study, family referral was predominant in the youngest age group, whereas self-referral was prominent in the 19–21 age group. In the 22–30 age group, self-referral and dentist referral were observed as the main modes of application. These findings suggest that awareness of orthodontic treatment need was particularly high among individuals aged 19–21. In addition, in the 22–30 age group, dentist referral was also noteworthy, alongside the high orthodontic treatment need.

Previous studies have reported that crowding in the anterior region is the most important factor motivating the desire to receive orthodontic treatment [22,38,39]. In our study, consistent with other studies, the strongest variable that maintained statistical robustness after Benjamini–Hochberg correction was crooked teeth (q = 0.018).

Studies show that most individuals need orthodontic treatment because of aesthetic concerns [35,40]. This pattern was also observed in our study. In addition, when evaluations of jaw position were examined, the most prominent difference was observed in the “I have no idea” response, which dropped from 60.0% (*n* = 24) in the 12–14 age group to 10.0% (*n* = 4) in the 19–21 age group. Concurrently, the assessment of “Normal” increased with age, rising from 20.0% in the 12–14 age group to 45.0% in both the 19–21 and 22–30 age groups. Among specific positional descriptions, “my upper jaw is forward” was most frequently reported in middle adolescence (15–18 years; 22.5%, *n* = 9), whereas “my lower jaw is backward” was most frequently reported in the 22–30 age group (17.5%, *n* = 7) (Supplementary Table S3). Overall, this distribution indicates that with increasing age, individuals are less likely to remain undecided and tend to either characterize their jaw relationship as normal or articulate a specific subjective directional perception. This observation may be interpreted strictly as a speculative hypothesis: early adolescence (12–14 years), when most participants reported no distinct perception of their jaw relationships, frequently coincides with the active growth phase during which skeletal discrepancies can be managed via functional orthopedic approaches. Conversely, by late adolescence, when individuals more readily recognize skeletal discrepancies, pubertal growth potential has likely largely ceased. From a theoretical clinical standpoint, this ‘delayed awareness’ could potentially limit the therapeutic window for functional interventions and, in theory, predispose patients to more invasive procedures, such as orthognathic surgery, later in life. On these grounds, educational initiatives aimed at raising awareness in younger age cohorts amenable to growth modification, alongside encouraging parents to seek early orthodontic consultations, are frequently emphasized in clinical practice. Nevertheless, because the present study utilized a cross-sectional design, these relationships were not directly tested within our cohort and must be regarded as a speculative clinical hypothesis rather than a definitive causal conclusion. Prospective longitudinal studies tracking cohorts from early adolescence through skeletal maturity are warranted to empirically evaluate whether a lack of early awareness genuinely impacts skeletal growth patterns and the subsequent need for surgical intervention in adulthood.

Among participants who wanted orthodontic treatment, dissatisfaction with dental appearance was the most frequent source of motivation in all age groups. The most pronounced difference was observed for the variable “environmental referral” (*p* < 0.001; *q* < 0.001; Cramer’s *V* = 0.438). The proportion of participants reporting this reason was 37.5% in the 12–14 age group and 50.0% in the 15–18 age group, whereas it decreased below 10% in the 19–21 and 22–30 age groups. This result shows that treatment desire in younger age groups is shaped more by external, familial, and social influences, whereas environmental referral decreases substantially at older ages. In addition, the rate at which others noticed participants’ dental irregularity increased markedly with age. It can be concluded that awareness of the need for orthodontic treatment increases not only among individuals but also among those around them; however, although such recognition becomes more common from the age of 19 onward, it does not translate into environmental referral.

The analyses demonstrating the main methodological distinction of the study can be interpreted as follows: the participant IOTN-AC category differed significantly between age groups (*p* < 0.001; Cramer’s *V* = 0.357). In participant assessments, the ‘no or slight need (1–4)’ category was observed at high rates of 62.5% in the 12–14 age group and 82.5% in the 15–18 age group, whereas this rate decreased to 25.0% in the 19–21 and 22–30 age groups. In contrast, perceptions of ‘moderate/borderline need (5–7)’ and ‘definite/great need (8–10)’ were observed more frequently in older age groups. No significant differences were found between age groups for the researcher IOTN-AC categories (Monte Carlo *p* = 0.972), IOTN-DHC grade (Monte Carlo *p* = 0.976), treatment need according to ICON (*p* = 0.822), or DAI category (Monte Carlo *p* = 0.506). The participant’s self-assessed IOTN-AC reflects purely subjective aesthetic perception and cannot be assumed to represent normative treatment need on its own. Conversely, researcher IOTN-AC demonstrated strong positive rank correlations with IOTN-DHC, DAI, and ICON (*ρ* = 0.755–0.861, all Holm-adjusted *p* < 0.001; Supplementary Table S5), confirming that researcher aesthetic scoring closely aligns with normative clinical criteria. Consequently, evaluating the discrepancy between participant and researcher ratings (ΔAC) serves as a valid proxy for awareness of normative treatment need rather than representing aesthetic concordance alone. This finding indicates that the researcher’s aesthetic evaluation was generally consistent with objective clinical indices. In our study, researcher–participant agreement differed across age groups (*p* < 0.001; Cramer’s *V* = 0.283), with higher levels of agreement between the researcher and participants’ self-perception observed in the young adolescent groups; substantially higher levels of concordance were observed in late adolescence and young adults, whereas exact concordance was rare in early adolescence (10.0%) and particularly sparse in middle adolescence (2.5%, *n* = 1). Because exact agreement was limited to a single participant in the 15–18-year cohort, parameter estimates for this specific group carry wide confidence intervals and are susceptible to sparse-data bias. Consequently, rather than demonstrating a discrete developmental turning point in middle adolescence, these findings indicate that substantial perceptual alignment with professional evaluations emerges primarily from late adolescence onward. The similarity we captured in normative treatment need suggests that the perceptual differences observed across age groups may reflect potential age-related tendencies in how malocclusion is subjectively perceived and evaluated by individuals, rather than distinct anatomical or morphological variations. The estimated prevalence of discordance, in turn, depended substantially on the operational threshold: 78.1% of participants differed from the researcher by at least one point, whereas 51.9% differed by more than one point. A one-point difference should therefore not automatically be interpreted as a clinically important perceptual deficit. Nevertheless, greater concordance in the two older groups persisted under both definitions. The ±1-point analysis is a sensitivity analysis, not an established minimal clinically important difference (MCID).

In the literature, it has been stated that rapid physical, psychological, and social changes occur during adolescence, making it difficult to determine what is normal and abnormal in this period [21]. As individuals progress through these developmental stages, they display distinct psychosocial behavioral patterns: consolidating body image, gaining emotional independence within the family, and assuming greater responsibility [21]. Adults have been reported to receive significantly less parental guidance regarding their orthodontic treatment [41]. This finding is consistent with developmental theories suggesting that autonomy increases with age and aligns with studies demonstrating that the influence of parents on orthodontic treatment decisions diminishes with age [41,42]. Consistent with these developmental patterns, family referral was predominant in younger age groups in our study, and difficulties in recognizing malocclusion were observed. In contrast, the transition to late adolescence, accompanied by greater emotional independence from the family and consolidation of body image, may be associated with awareness that is more concordant with professional evaluations.

### Limitations

The limitations of this study include its cross-sectional design, the use of non-probability convenience sampling, and recruitment from a single center. Because this non-probability approach may introduce an inherent self-selection bias, it likely contributed to higher observed awareness and aesthetic concern levels than would be expected in the broader, untreated general population, thereby limiting direct generalizability. Specifically, because our cohort was recruited exclusively from individuals presenting to an orthodontic clinic, this setting likely introduced an upward bias in the observed rates of concordant perception and treatment awareness compared to what would be expected in an unselected, non-clinical population. However, although the prespecified 1:1 sex-stratified quota design minimized sex-related confounding across age groups, it created an artificial balance that may not fully reflect the natural distribution, thereby presenting an additional constraint on external generalizability. Furthermore, the single-center design inherently limits external validity. Participants seeking care at a single university clinic may present with clinical characteristics, aesthetic expectations, and socioeconomic profiles that differ from patients attending other regional centers or private practices; they may also differ fundamentally from individuals in the general population who never seek orthodontic consultation due to a lack of awareness, financial constraints, or geographical barriers. Additionally, because our institution operates as a regional tertiary academic referral hospital in Southeastern Türkiye, the attending patient profile reflects distinct sociodemographic characteristics and potentially more severe, complex malocclusions compared to individuals treated in secondary dental clinics, private practices, or other geographical settings. Consequently, our findings cannot be directly extrapolated to community-level awareness and should not be used uncritically for public health planning. Another statistical limitation concerns sparse outcome cells; specifically, the small number of participants with overestimation (*n* = 14) and the presence of only a single concordant participant in the 15–18 age group yielded wide confidence intervals. Combining the two youngest groups increased the concordant reference count to five but did not eliminate sparse-data bias, leaving pooled estimates exploratory. Similarly, in the ±1-point sensitivity analysis, sparse outcome cells (*n* = 5 in the overestimation group with a zero cell) precluded multinomial logistic regression modeling. Although model reduction and age-only sensitivity models supported the observed trend, they do not entirely eliminate the potential for sparse-data bias or statistical imprecision. However, evaluating all developmental groups under identical clinical conditions and on the same baseline framework provided methodological standardization across groups. This approach minimized potential confounding variables when comparing awareness levels across developmental periods, allowing for a standardized comparison among groups. Nevertheless, due to the cross-sectional design, the same participants could not be followed longitudinally over time, which prevented individual changes from being clearly demonstrated.

Despite these limitations, our study has several notable strengths. First, the combined use of IOTN, DAI, and ICON enabled a comprehensive, multidimensional evaluation of orthodontic treatment need from both objective and subjective perspectives using internationally validated scales rather than relying on a single index, while also establishing a robust normative benchmark to validate the researcher’s aesthetic assessments. In addition, the face-to-face clinical completion of all evaluation forms by the same researcher ensured a high degree of measurement standardization and eliminated interobserver variation. We recommend that future studies take into account the stated limitations of both our study and the previous literature.

## 6. Conclusions

The results of this study, in which participants were grouped according to developmental periods, are as follows:

1. The desire to receive orthodontic treatment remained high across all evaluated age cohorts, being slightly more pronounced during adolescence than in young adults.
2. Personal awareness of morphological and skeletal discrepancies strengthens with age, with treatment-need underestimation diminishing significantly from age 19 onward in favor of concordant perception. However, conclusions specific to middle adolescence remain uncertain because exact concordance was sparse.
3. Researcher IOTN-AC aesthetic evaluations demonstrated strong rank alignment with established objective clinical indices (IOTN-DHC, DAI, and ICON), indicating that the observed score discrepancy may serve as an informative proxy for awareness of normative treatment need rather than representing mere aesthetic concordance.

In light of these findings, it may be proposed as a clinical hypothesis that enhancing awareness of orthodontic treatment need in younger age cohorts could facilitate timely access to appropriate intervention. Nevertheless, given the cross-sectional design of this study, prospective longitudinal research is warranted to directly evaluate this hypothesis and determine the long-term clinical consequences and treatment-timing implications of early awareness deficits.

## Figures and Tables

**Table 1:** Scales and assessment methods used.

Assessment Form	Scale Used	Evaluator	Purpose of Assessment
Additional to the questionnaire	IOTN-AC	Participant	Evaluation of orthodontic treatment need according to the participant’s own perception
Additional to the questionnaire	VAS	Participant	Evaluation of dental aesthetic perception according to the participant’s own perception
Participant evaluation form	IOTN-AC	Researcher	Evaluation of orthodontic treatment need according to the expert orthodontist’s perception
Participant evaluation form	VAS	Researcher	Evaluation of dental aesthetic perception according to the expert orthodontist’s perception *
Participant evaluation form	IOTN-DHC	Researcher	Objective evaluation of orthodontic treatment need
Participant evaluation form	ICON	Researcher	Objective evaluation of orthodontic treatment need
Participant evaluation form	DAI	Researcher	Objective evaluation of orthodontic treatment need

The IOTN-AC and VAS scales included in the participant evaluation form are identical to the IOTN-AC and VAS scales completed by the participants as part of the questionnaire. * Researcher-assessed VAS was not included in any statistical hypothesis testing or multivariable regression models to prevent analytical redundancy.

**Table 2:** Distribution of participants’ sex, previous orthodontic application history, and mode of application to the clinic according to age groups.

Variable	12–14 Years	15–18 Years	19–21 Years	22–30 Years	Total	*p*	Effect Size
Female sex	20 (50.0)	20 (50.0)	20 (50.0)	20 (50.0)	80 (50.0)	1.000	Cramer’s *V* = 0.000
Previous orthodontic application history, yes	5 (12.5)	5 (12.5)	16 (40.0)	23 (57.5)	49 (30.6)	<0.001	Cramer’s *V* = 0.416
Mode of application to clinic						<0.001	Cramer’s *V* = 0.446
└ Self-referral	9 (22.5)	8 (20.0)	31 (77.5)	21 (52.5)	69 (43.1)		
└ Family referral	22 (55.0)	9 (22.5)	3 (7.5)	0 (0.0)	34 (21.2)		
└ Dentist referral	7 (17.5)	12 (30.0)	6 (15.0)	19 (47.5)	44 (27.5)		
└ Friend referral	1 (2.5)	11 (27.5)	0 (0.0)	0 (0.0)	12 (7.5)		
└ Teacher referral	1 (2.5)	0 (0.0)	0 (0.0)	0 (0.0)	1 (0.6)		

Data are *n* (%), with column percentages. Pearson’s chi-square test was used for sex (reflecting the fixed 1:1 stratified quota design across cohorts) and previous attendance; the mode of application comparison used the Fisher–Freeman–Halton test with Monte Carlo estimation (1,000,000 samples; seed 20261001). Cramer’s *V* is based on Pearson’s chi-square statistic.

**Table 5:** Exploratory multinomial logistic regression of raw-score agreement by age group and sex.

Covariate	Underestimation vs. Concordant Perception OR (95% CI)	*p*	Overestimation vs. Concordant Perception OR (95% CI)	*p*
Age group: 15–18 years (ref: 12–14)	4.37 (0.46–41.14)	0.197	4.00 (0.27–60.41)	0.316
Age group: 19–21 years (ref: 12–14)	0.19 (0.06–0.65)	0.008	0.38 (0.06–2.45)	0.309
Age group: 22–30 years (ref: 12–14)	0.15 (0.04–0.52)	0.003	0.33 (0.05–2.12)	0.244
Male sex (ref: female)	1.31 (0.57–3.00)	0.526	1.22 (0.35–4.28)	0.758

The reference outcome is concordant perception (*n* = 35); underestimation *n* = 111 and overestimation *n* = 14. Covariates are age group and sex only. The model has four slope coefficients per contrast (10 parameters including two intercepts). ORs compare each outcome with concordance, not risks or probabilities. Wald 95% CIs are reported. VAS, researcher IOTN-AC category, and previous attendance were omitted in the reduced model. The age-group-only sensitivity model is reported in Supplementary Table S8. Estimates are exploratory and imprecise for sparse outcome cells. OR, odds ratio; CI, confidence interval; ref, reference.

## Data Availability

The original contributions presented in the study are included in the article; further information is available from the corresponding author upon reasonable request.

## Supplementary Materials

The following supporting information can be downloaded at: https://www.mdpi.com/article/10.3390/healthcare14203501/s1. Supplementary Table S1. Comparison of subjective awareness, aesthetic/skeletal perception, and subjective orthodontic treatment evaluations according to age groups. Supplementary Table S2. Distribution of reasons suggesting the necessity of orthodontic treatment according to age groups. Supplementary Table S3. Distribution of participants’ subjective evaluations of jaw position according to age groups. Supplementary Table S4. Distribution of reasons for wanting orthodontic treatment according to age groups. Supplementary Table S5. Benchmarking of researcher raw IOTN-AC against clinical indices. Supplementary Table S6. Analysis of awareness agreement between participant and researcher IOTN-AC assessments using a ±1-point tolerance margin. Supplementary Table S7. Comparison of clinical and social awareness characteristics according to treatment-need awareness agreement. Supplementary Table S8. Age-group-only multinomial sensitivity analysis. Supplementary Table S9. Sensitivity analysis combining the 12–14- and 15–18-year groups.

## Abbreviations

The following abbreviations are used in this manuscript:

BH-FDR	Benjamini–Hochberg False Discovery Rate
CI	Confidence Intervals
DAI	Dental Aesthetic Index
ICON	Index of Complexity, Outcome and Need
IOTN	Index of Orthodontic Treatment Need
IOTN-AC	Index of Orthodontic Treatment Need-Aesthetic Component
IOTN-DHC	Index of Orthodontic Treatment Need-Dental Health Component
MCID	Minimal Clinically Important Difference
OR	Odds Ratios
SD	Standard Deviation
VAS	Visual Analog Scale
ΔAC	Aesthetic Component Discrepancy (Participant minus Researcher)
