# Supplementary material for: Evaluation of Awareness of the Need for Orthodontic Treatment Across Different Age Groups in Adolescents and Young Adults
Source: Healthcare (Basel). 2026 Oct 18;14(20):3501. doi: 10.3390/healthcare14203501 (PMC13704060; doi:10.3390/healthcare14203501)
Supplement: Supplementary file 1 [file healthcare-14-03501-s001.zip › healthcare-4598622-supplementary.pdf]

SUPPLEMENTARY TABLES

Supplementary Table S1. Comparison of subjective awareness, aesthetic/skeletal perception, and subjective orthodontic treatment evaluations according to age groups

**Variable**	**12–14 years**	**15–18 years**	**19–21 years**	**22–30 years**	**Total**	***p***	***q* (BH-FDR)**	**Effect size**	**Test**
Dissatisfaction with dental appearance	28 (70.0)	34 (85.0)	34 (85.0)	27 (67.5)	123 (76.9)	0.111	0.200	Cramer's V=0.194	Pearson
Noticing irregularity in one's own teeth	29 (72.5)	31 (77.5)	38 (95.0)	38 (95.0)	136 (85.0)	0.005	0.014	Cramer's V=0.284	Pearson
Thinking that tooth position should change for facial/lip aesthetics	26 (65.0)	27 (67.5)	26 (65.0)	17 (42.5)	96 (60.0)	0.076	0.171	Cramer's V=0.207	Pearson
Noticing facial asymmetry/deviation	7 (17.5)	10 (25.0)	7 (17.5)	9 (22.5)	33 (20.6)	0.794	0.893	Cramer's V=0.080	Pearson
Desire for orthodontic treatment	32 (80.0)	34 (85.0)	34 (85.0)	27 (67.5)	127 (79.4)	0.172	0.258	Cramer's V=0.177	Pearson
Thinking orthodontic treatment is necessary	37 (92.5)	38 (95.0)	38 (95.0)	38 (95.0)	151 (94.4)	1.000	1.000	Cramer's V=0.047	FFH exact
Thinking one has a skeletal jaw discrepancy	5/40 (12.5)	8/40 (20.0)	19/40 (47.5)	17/39 (43.6)	49/159 (30.8)	<0.001	0.004	Cramer's V=0.324	Pearson
Others noticing dental irregularity: yes	10 (25.0)	18 (45.0)	35 (87.5)	37 (92.5)	100 (62.5)	<0.001	<0.001	Cramer's V=0.588	Pearson
Participant-assessed Visual Analog Scale (VAS) score (mm), mean±SD	52.55 ± 21.66	50.92 ± 26.98	46.02 ± 20.86	48.60 ± 22.18	49.52 ± 22.96	0.611	0.785	η^2=0.012	ANOVA

Data are presented as n (%) or n/N (%) for categorical variables and mean±SD for the VAS score. Pearson's chi-square test was used for categorical variables; when expected cell count assumptions were not met, the Fisher–Freeman–Halton exact test was used. Participant-assessed VAS was analyzed using one-way ANOVA. BH-FDR adjustment was applied across all nine comparisons in Supplementary Table S1. One skeletal-item response was missing in the oldest age group; therefore, the overall denominator was 159. η^2 denotes ANOVA eta-squared; BH-FDR, Benjamini–Hochberg false discovery rate; SD, standard deviation; FFH, Fisher–Freeman–Halton.

Supplementary Table S2. Distribution of reasons suggesting the necessity of orthodontic treatment according to age groups (among participants who considered treatment necessary, *n*=151)

**Variable**	**12–14 years** (*n*=37)	**15–18 years** (*n*=38)	**19–21 years** (*n*=38)	**22–30 years** (*n*=38)	**Total** (*n*=151)	***p***	***q* (BH-FDR)**	**Effect size**	**Test**
Crowding	16 (43.2)	20 (52.6)	24 (63.2)	29 (76.3)	89 (58.9)	0.024	0.067	Cramer's *V*=0.250	Pearson
Spacing between teeth	8 (21.6)	12 (31.6)	7 (18.4)	4 (10.5)	31 (20.5)	0.151	0.237	Cramer's *V*=0.187	Pearson
Crooked teeth	17 (45.9)	21 (55.3)	6 (15.8)	11 (28.9)	55 (36.4)	0.002	0.018	Cramer's *V*=0.317	Pearson
Some teeth positioned higher	11 (29.7)	17 (44.7)	10 (26.3)	6 (15.8)	44 (29.1)	0.048	0.088	Cramer's *V*=0.229	Pearson
Upper anterior teeth positioned forward	9 (24.3)	17 (44.7)	8 (21.1)	6 (15.8)	40 (26.5)	0.024	0.067	Cramer's *V*=0.249	Pearson
Upper anterior teeth positioned backward	2 (5.4)	5 (13.2)	5 (13.2)	4 (10.5)	16 (10.6)	0.681	0.681	Cramer's *V*=0.102	FFH exact
Missing tooth	5 (13.5)	4 (10.5)	3 (7.9)	7 (18.4)	19 (12.6)	0.552	0.607	Cramer's *V*=0.118	FFH exact
Supernumerary tooth	4 (10.8)	8 (21.1)	4 (10.5)	0 (0.0)	16 (10.6)	0.016	0.067	Cramer's *V*=0.243	FFH exact
Jaw/jaws position disorder	5 (13.5)	13 (34.2)	12 (31.6)	10 (26.3)	40 (26.5)	0.182	0.250	Cramer's *V*=0.180	Pearson
Narrow jaw	7 (18.9)	10 (26.3)	7 (18.4)	13 (34.2)	37 (24.5)	0.336	0.410	Cramer's *V*=0.150	Pearson
Dissatisfaction with facial appearance	8 (21.6)	14 (36.8)	19 (50.0)	10 (26.3)	51 (33.8)	0.046	0.088	Cramer's *V*=0.230	Pearson

Data are presented as n (%). Denominators represent the conditional subgroup of participants who stated "I think orthodontic treatment is necessary" and answered the follow-up items on perceived irregularities ($n = 151$; 12–14 years: $n = 37$; 15–18 years: $n = 38$; 19–21 years: $n = 38$; 22–30 years: $n = 38$), rather than the total sample ($N = 160$). Percentages are calculated using these subgroup denominators. Tests are identified in the final column; FFH exact denotes full fixed-margin enumeration. BH-FDR correction was applied across all 11 comparisons. Cramer's V was calculated from Pearson's chi-square statistic. BH-FDR, Benjamini–Hochberg false discovery rate; FFH, Fisher–Freeman–Halton.

Supplementary Table S3. Distribution of participants' subjective evaluations of jaw position according to age groups

**Opinion about the position of the jaws**	**12-14 years**	**15-18 years**	**19-21 years**	**22-30 years**	**Total**
Normal	8 (20.0)	12 (30.0)	18 (45.0)	18 (45.0)	56 (35.0)
My upper jaw is forward	3 (7.5)	9 (22.5)	5 (12.5)	4 (10.0)	21 (13.1)
My upper jaw is backward	0 (0.0)	0 (0.0)	1 (2.5)	1 (2.5)	2 (1.2)
My lower jaw is forward	3 (7.5)	1 (2.5)	6 (15.0)	3 (7.5)	13 (8.1)
My lower jaw is backward	2 (5.0)	4 (10.0)	4 (10.0)	7 (17.5)	17 (10.6)
Upper forward + lower backward	0 (0.0)	0 (0.0)	2 (5.0)	0 (0.0)	2 (1.2)
I have no idea	24 (60.0)	14 (35.0)	4 (10.0)	7 (17.5)	49 (30.6)

One omnibus Fisher–Freeman–Halton test with Monte Carlo estimation was used ($p<0.001$; 1,000,000 samples; seed 20261001). Cramer's $V=0.301$ was calculated from Pearson's chi-square statistic and is reported as an effect size, not as a second significance test. Multiple responses were allowed; the observed combined upper-forward/lower-backward response was retained as a single category.

Supplementary Table S4. Distribution of reasons for wanting orthodontic treatment according to age groups (among participants who wanted orthodontic treatment, *n*=127)

**Variable**	**12–14 years** (*n*=32, 80.0%)	**15–18 years** (*n*=34, 85.0%)	**19–21 years** (*n*=34, 85.0%)	**22–30 years** (*n*=27, 67.5%)	**Total** (*n*=127, 79.4%)	***p***	***q* (BH-FDR)**	**Effect size**	**Test**
Dissatisfaction with dental appearance	25 (78.1)	31 (91.2)	32 (94.1)	23 (85.2)	111 (87.4)	0.234	0.234	Cramer's *V*=0.187	FFH exact
Dissatisfaction with jaw appearance	7 (21.9)	11 (32.4)	16 (47.1)	12 (44.4)	46 (36.2)	0.133	0.187	Cramer's *V*=0.210	Pearson
Desire to chew comfortably	6 (18.8)	15 (44.1)	14 (41.2)	13 (48.1)	48 (37.8)	0.075	0.156	Cramer's *V*=0.233	Pearson
Desire to swallow comfortably	4 (12.5)	11 (32.4)	6 (17.6)	8 (29.6)	29 (22.8)	0.178	0.208	Cramer's *V*=0.197	Pearson
Desire to breathe comfortably	2 (6.2)	12 (35.3)	2 (5.9)	7 (25.9)	23 (18.1)	0.002	0.008	Cramer's *V*=0.336	FFH exact
Desire to improve speech	7 (21.9)	12 (35.3)	4 (11.8)	4 (14.8)	27 (21.3)	0.089	0.156	Cramer's *V*=0.226	Pearson
Environmental referral	12 (37.5)	17 (50.0)	2 (5.9)	2 (7.4)	33 (26.0)	<0.001	<0.001	Cramer's *V*=0.438	Pearson

Multiple-response items were evaluated among participants who wanted treatment (n=127/160, 79.4%; cohort-specific desire rates: 32/40 (80.0%), 34/40 (85.0%), 34/40 (85.0%), and 27/40 (67.5%), respectively). Percentages may sum to more than 100%. Thirty-three non-applicable cases were excluded. Tests are identified in the final column. BH-FDR correction was applied across the seven comparisons. FFH exact denotes full fixed-margin enumeration; Cramer's V is based on Pearson's chi-square statistic. BH-FDR, Benjamini-Hochberg false discovery rate; FFH, Fisher–Freeman–Halton.

Supplementary Table S5. Benchmarking of researcher raw IOTN-AC against clinical indices.

Clinical index	*N*	Spearman *ρ*	95% CI	Holm-adjusted *p*
IOTN-DHC grade	160	0.861	0.804–0.904	< 0.001
DAI total	160	0.755	0.664–0.827	< 0.001
ICON total	160	0.860	0.802–0.903	< 0.001

Researcher raw IOTN-AC was compared with recorded IOTN-DHC grade and continuous DAI and ICON totals. The 95% CIs are percentile intervals from 20,000 paired bootstrap resamples per comparison (seeds 20261018, 20261019, and 20261020, respectively). Two-sided asymptotic p values were Holm-adjusted across the three correlations. Correlation quantifies association, not interchangeability or diagnostic accuracy. ICON incorporates an IOTN-AC aesthetic component and therefore is not an independent reference for validating IOTN-AC. All measurements were complete. IOTN-DHC, Index of Orthodontic Treatment Need–Dental Health Component; DAI, Dental Aesthetic Index; ICON, Index of Complexity, Outcome and Need; CI, confidence interval.

Supplementary Table S6. Analysis of awareness agreement between participant and researcher IOTN-AC assessments using a ±1-point tolerance margin.

Age group (years)	*N*	Underestimation $\Delta AC < -1$	Concordance $-1 \le \Delta AC \le 1$	Overestimation $\Delta AC > 1$
12–14	40	27 (67.5)	10 (25.0)	3 (7.5)
15–18	40	32 (80.0)	7 (17.5)	1 (2.5)
19–21	40	8 (20.0)	32 (80.0)	0 (0.0)
22–30	40	11 (27.5)	28 (70.0)	1 (2.5)
Total	160	78 (48.8)	77 (48.1)	5 (3.1)

Data are n (%) within age group; total percentages use $N = 160$. ΔAC is the participant minus researcher raw IOTN-AC score. The four-group Fisher–Freeman–Halton test used 1,000,000 fixed-margin Monte Carlo samples, probability ordering, and a plus-one correction (seed 20261009). Monte Carlo $p < 0.001$; 0 of 1,000,000 samples were at least as extreme as the observed table; estimated $p = 1.00 \times 10^{-6}$, with a binomial 95% Monte Carlo interval of 0 to 3.69×10^{-6}. Cramer's $V = 0.395$, calculated from Pearson's chi-square statistic. The original exact-concordance proportion was 35/160 (21.9%). A multinomial model was not fitted because overestimation was sparse and included a zero cell. IOTN-AC, Index of Orthodontic Treatment Need–Aesthetic Component. CI, confidence interval; N/n, number of participants; ΔAC, difference in raw Aesthetic Component score.

Supplementary Table S7. Comparison of clinical and social awareness characteristics according to treatment-need awareness agreement

Variable	Underestimation	Concordance	Overestimation	Total	*p*	Effect size	Post-hoc	Test
Previous orthodontic application history, yes	29/111 (26.1)	17/35 (48.6)	3/14 (21.4)	49/160 (30.6)	0.038	Cramer's *V*=0.208		FFH exact
Mode of application to clinic					<0.001	Cramer's *V*=0.279		FFH MC
└ Self-referral	38 (34.2)	24 (68.6)	7 (50.0)	69 (43.1)				
└ Family referral	31 (27.9)	1 (2.9)	2 (14.3)	34 (21.2)				
└ Dentist referral	30 (27.0)	9 (25.7)	5 (35.7)	44 (27.5)				
└ Friend referral	12 (10.8)	0 (0.0)	0 (0.0)	12 (7.5)				
└ Teacher referral	0 (0.0)	1 (2.9)	0 (0.0)	1 (0.6)				
Dissatisfaction with dental appearance	84/111 (75.7)	28/35 (80.0)	11/14 (78.6)	123/160 (76.9)	0.910	Cramer's *V*=0.044		FFH exact
Noticing irregularity in one's own teeth	89/111 (80.2)	34/35 (97.1)	13/14 (92.9)	136/160 (85.0)	0.026	Cramer's *V*=0.205		FFH exact
Thinking one has a skeletal jaw discrepancy	25/110 (22.7)	21/35 (60.0)	3/14 (21.4)	49/159 (30.8)	<0.001	Cramer's *V*=0.336		FFH exact
Others noticing dental irregularity	58/111 (52.3)	32/35 (91.4)	10/14 (71.4)	100/160 (62.5)	<0.001	Cramer's *V*=0.335		FFH exact
Participant-assessed VAS score, mm, median [IQR]	50.0 [38.5-69.0]	40.0 [22.0-46.5]	47.0 [37.2-68.2]	47.0 [36.0-66.0]	0.007	ε^2=0.062	Concordant vs underestimation: *p*=0.005	Kruskal–Wallis

Data are presented as n (%) or n/N (%) for categorical variables and median [Q1, Q3] for VAS. Agreement groups are defined based on the sign of the participant-minus-researcher raw IOTN-AC score difference: negative ($\Delta AC < 0$), zero ($\Delta AC = 0$), and positive ($\Delta AC > 0$). Denominators vary slightly for perceived skeletal jaw discrepancy (valid $n = 110$ in the underestimation group; total valid $N = 159$) due to an isolated recording omission ($n = 1$) during the investigator-administered face-to-face questionnaire; percentages are calculated based on valid responses. All other rows include 111, 35, and 14 participants. FFH exact denotes full enumeration; FFH MC denotes Fisher–Freeman–Halton Monte Carlo estimation (1,000,000 samples; seed 20261001). The p value for multicategory items refers to the overall variable distribution, not individual subcategory rows. Participant-assessed VAS was analyzed using the Kruskal–Wallis test with Dunn–Bonferroni pairwise comparisons. Rank $\varepsilon^2 = H/(N-1)$. IQR, interquartile range; ε^2, epsilon-squared; FFH, Fisher–Freeman–Halton.

Supplementary Table S8. Age-group-only multinomial sensitivity analysis

Covariate	Underestimation vs concordance OR (95% CI)	*p*	Overestimation vs concordance OR (95% CI)	*p*
15–18 years (ref: 12–14)	4.36 (0.46–41.06)	0.198	4.00 (0.27–60.32)	0.317
19–21 years (ref: 12–14)	0.19 (0.06–0.66)	0.009	0.38 (0.06–2.46)	0.310
22–30 years (ref: 12–14)	0.15 (0.04–0.52)	0.003	0.33 (0.05–2.13)	0.246

Reference outcome: exact raw-score concordance; reference age: 12–14 years. N=160. The model has six slopes and two intercepts. Wald confidence intervals are exploratory; sparse-data bias and imprecision remain possible. This model was specified as a reviewer-requested sensitivity analysis.

Supplementary Table S9. Sensitivity analysis combining the 12–14- and 15–18-year groups.

A. Exact raw-score outcome distribution

Age group (years)	*N*	Underestimation<br>ΔAC < 0	Exact concordance<br>ΔAC = 0	Overestimation<br>ΔAC > 0
12–18	80	69 (86.3)	5 (6.3)	6 (7.5)
19–21	40	22 (55.0)	14 (35.0)	4 (10.0)
22–30	40	20 (50.0)	16 (40.0)	4 (10.0)

Data are n (%) within age group. Fisher–Freeman–Halton Monte Carlo p < 0.001 (1,000,000 fixed-margin samples; seed 20261010; probability ordering; plus-one estimate 1.30×10^{-5}; binomial 95% Monte Carlo interval 6.20×10^{-6} to 2.10×10^{-5}). Cramer's V = 0.280.

B. Exploratory sex-adjusted multinomial model with exact concordance as the outcome reference

Covariate	Underestimation<br>OR (95% CI)	*p*	Overestimation<br>OR (95% CI)	*p*
19–21 vs. 12–18 years	0.11 (0.04–0.35)	< 0.001	0.24 (0.05–1.21)	0.083
22–30 vs. 12–18 years	0.09 (0.03–0.28)	< 0.001	0.21 (0.04–1.04)	0.056
Male vs. female	1.31 (0.57–2.99)	0.528	1.22 (0.35–4.28)	0.759

N = 160; 111 underestimation, 35 exact concordance, and 14 overestimation. The pooled 12–18-year reference group contained only five exactly concordant participants. The model estimated three slopes per contrast and two intercepts (eight parameters in total); maximum-likelihood estimation converged. Confidence intervals are Wald intervals. Estimates are exploratory. OR, odds ratio; CI, confidence interval.
